# The Relationship between Korean Adolescents’ Sports Participation, Internal Health Locus of Control, and Wellness during COVID-19

**DOI:** 10.3390/ijerph18062950

**Published:** 2021-03-13

**Authors:** Dae-Jung Lee, Wi-Young So, Seung-Man Lee

**Affiliations:** 1 Department of Physical Education, Jeonbuk National University, Jeollabuk-do 54896, Korea; dleownd23@hanmail.net; 2 Sports Medicine Major, College of Humanities and Arts, Korea National University of Transportation, Chungju-si 27469, Korea; wowso@ut.ac.kr; 3 Department of Physical Education, College of Education, Korea University, Seoul 02841, Korea

**Keywords:** adolescent, internal health locus of control, sports participation, structural equation modeling, wellness

## Abstract

This study was aimed at providing practical information to improve Korean adolescents’ wellness by empirically identifying its correlation with sports participation and having an internal health locus of control (IHLC) during the COVID-19 pandemic. This study comprised both a pilot test and a main study. We recruited 844 Korean adolescents as subjects in January 2021 to participate in an online self-reported survey. The reliability and validity of the scales used (sports participation, IHLC, and wellness) were verified through a pilot test. In the main study, we verified the differences between all variables according to adolescents’ demographic characteristics and the structural relationship of sports participation, IHLC, and wellness. Sports participation had a positive effect on IHLC (*p* < 0.001) and wellness (*p* < 0.001). Additionally, IHLC had a positive effect on wellness (*p* < 0.001). In juvenile educational institutions, there is a need to develop strategies to increase wellness, sports participation, and IHLC among adolescent students, which can improve their wellness in the context of the COVID-19 pandemic.

## 1. Introduction

With the global spread of the coronavirus disease (COVID-19), the World Health Organization (WHO) identified COVID-19 as a “public health emergency of international concern” on 30 January 2020, and declared it a pandemic on 11 March—the highest level of risk for infectious diseases. As a result, people faced unprecedented restrictions worldwide, particularly that of social distancing. While it is one of the most effective measures to prevent COVID-19 spread, social distancing poses its own risks for mental health. Therefore, COVID-19 poses both direct and indirect risks to health. For instance, Nsearchou et al. [1] found that fear and worry about contracting COVID-19 caused stress, depression, anxiety, obsessive symptoms, somatic symptoms, and intensified behavioral problems, which could negatively affect the health of young people. Further, according to O’Connor et al. [2], suicidal thoughts among young adults increased in the initial weeks of social distancing, with one in seven (14%) reporting suicidal ideation. Though not directly comparable to the pre-COVID-19 period, the rate of suicidal thoughts among young adults during the COVID-19 pandemic (12.5–14.4%) was higher than that reported in another study before the pandemic (11%) [3].

Moreover, a longer stay at home due to social distancing can lead to increased sedentary behavior (such as playing games, watching television, and using mobile devices) and the reduction or avoidance of regular physical activity (PA), which can result in chronic health risks and deterioration [4]. A recent report by the WHO [5] found that most adolescents do not even exercise for 60 min a day, as recommended by the WHO, suggesting that their long-term health is at risk. Therefore, it is time for governments to focus on the mental and physical health of adolescents and find ways to offset the harmful effects of social distancing and isolation [6].

Personal health determinants can be largely classified into heredity, environment, and behavior. Among them, environment and behavior can be controlled [7] through individual choices. Health has traditionally been understood as a complete physical, mental, and social condition [8]. Recently, however, the conventional concept of health has been expanded into a more active and comprehensive concept, resulting in the emergence of the concept of “wellness.” Wellness is defined as an individual’s physical, mental, social, and spiritual health, obtained by maintaining a harmonious and balanced life and improving quality of life [9]. Wellness includes active efforts to maximize one’s potential and active processes to enrich and beautify one’s life.

Wellness and sports participation are closely related. For example, it has been reported that the health benefits of sports participation extend to mental and social factors [10], in addition to its well-known physical health benefits, such as improving bone density, aerobic physical fitness, muscular strength, and reducing cardiovascular disease risk [11]. Moreover, basic PA is associated with strengthening the immune system [12] and has the potential to prevent respiratory infections [13]. Consequently, regular, medium-intensity physical exercise (carried out indoors, outdoors, or at home) appears to be essential for anyone affected by COVID-19 [14].

In this study, we focus on the internal health locus of control (IHLC) as a factor that can affect wellness. The health locus of control (HLC) is defined as the extent to which individuals believe that health is the result of their own actions, luck, or other influences. The HLC has three categories: IHLC, powerful others health locus of control (PHLC), and chance health locus of control (CHLC). Individuals with an IHLC believe that health outcomes are attributable to individual behavior and self-control; those with PHLC mainly identify external causes, such as other people, as determinants of their health; and individuals with CHLC believe that their health is determined by circumstantial/accidental factors [15]. However, according to Bennett et al. [16], people with a strong IHLC and PHLC use smartphone applications to take an active role in their health, while those with CHLC tendencies do not. In particular, Croster et al. [17] found that people lacking an IHLC tend to experience emotional depletion and discomfort, while those with high levels can expect their IHLC to protect them from COVID-19-induced stressors.

Previous studies have shown that sports participation and having an IHLC influence each other. Helmer et al. [18] found that college students with a strong IHLC paid more attention to healthy nutrition and reported higher levels of sports participation. Further, Adame et al. [19] reported that health was significantly related to the amount of exercise and the IHLC. Other studies have shown that wellness is affected by having an IHLC [20]. As such, the preceding study reported meaningful results on the relationship between sports participation, health control and care, and wellness [18,19,20]. However, most studies have failed to examine the relationship among all variables comprehensively. In addition, most studies have focused on patients with diseases [21,22], adults [23,24], and college students [18,25], while there is a lack of studies on adolescents, who are in a critical period of learning and growth.

To overcome this gap in the existing literature, we aim to verify the overall relationship among sports participation, IHLC, and wellness at a time when global interest in health is increasing due to the COVID-19 pandemic. This is an opportune time for the present study, as youth sports participation, IHLC, and wellness are expected to show marked differences in the context of the pandemic. Even with the development of vaccines to prevent COVID-19, it could still take a long time for the pandemic to end. Therefore, measures should be taken to maintain and strengthen the health of teenagers, with a focus on personal hygiene. Accordingly, this study aims to provide practical information to enhance the wellness of adolescents in the context of COVID-19 by empirically identifying the relationship between sports participation, IHLC, and wellness. Therefore, in this study, we developed the following hypotheses: first, that there are differences between IHLC and wellness depending on the frequency, intensity, and duration of sports participation (Hypothesis 1); second, that sports participation has a positive effect on IHLC (Hypothesis 2); third, that sports participation has a positive effect on wellness (Hypothesis 3); and fourth, that IHLC has a positive effect on wellness (Hypothesis 4).

## 2. Materials and Methods

### 2.1. Participants

In total, 844 Korean adolescents studying in either middle school or high school were recruited through convenience sampling. Among them, 212 adolescents were selected for the preliminary survey, and 632 were selected for the main survey. To collect data, we conducted an online self-reported survey (presented in Google Forms) from January 7 to January 15, 2021. This study was conducted after obtaining ethical approval from the Institutional Review Board of Jeonbuk National University (JBNU 2020-11-010-001). The demographic characteristics of participants are shown in Table 1.

### 2.2. Instruments

Items regarding demographic variables collected data regarding participants’ sex as well as the frequency, intensity, and duration of their sports participation, which were considered to be closely related to IHLC and wellness variables.

Items related to sports participation were based on the sports participation classification model of Snyder and Spreitzer [26], using measures that verified reliability and validity in Lee et al. [27]. Sports participation consists of three sub-dimensions: cognitive participation, behavioral participation, and affective participation. To assess HLC, we used a questionnaire developed by Wallston et al. [28]. HLC consists of three sub-dimensions: internal, external, and luck, but only six items of HLC were used as a single variable to meet the purpose of this study. Anspaugh et al. [29] modified and supplemented the wellness questionnaire to be used for adolescents. Wellness consists of five sub-dimensions: physical, social, mental, intellectual, and emotional health. Sports participation, IHLC, and wellness were scored on a five-point Likert scale, ranging from 5 (very much so) to 1 (not at all).

### 2.3. Reliability and Validity of the Instruments

To verify the reliability of the items utilized in this study, Cronbach’s α was used to check their internal consistency, while confirmatory factor analysis (CFA) was used to verify their validity. The Cronbach’s α for all observed variables ranged from 0.633 to 0.938, all of which were above the baseline of 0.6, which indicated that all variables exhibited high internal consistency. However, the “alpha if item deleted” was higher than “Cronbach’s α,” indicating higher reliability when the item was removed. Thus, the study was conducted after deleting the two corresponding questions (mental health #1 and intellectual health #1).

The proposed model’s fit is shown as follows: root mean square residual (RMR) = 0.050, normed fit index (NFI) = 0.814, incremental fit index (IFI) = 0.851, comparative fit index (CFI) = 0.849, root mean square error of approximation (RMSEA) = 0.125, and did not meet the baseline. Thus, some observed variables (mental health) were eliminated based on their squared multiple correlation (SMC) values. As a result, the goodness-of-fit of the modified model can be assessed as acceptable as RMR = 0.042, NFI = 0.902, IFI = 0.931, CFI = 0.931, RMSEA = 0.099.

Next, the validity of the model was verified according to the CFA results. First, three methods were used to verify convergent validity, namely, standardized regression coefficient, average variance extraction (AVE), and construct reliability (CR). The results of the CFA are shown in Table 2. The range of standardized regression coefficients for all variables lay between 0.571 and 0.922, while the significance (critical ratio) was 1.965 or higher. Furthermore, the CR was found to be 0.980–0.986, and the AVE was 0.906–0.958, which satisfied all three conditions to secure convergent validity.

Next, the correlations of the constructs and AVE were compared to verify the discriminant validity. The results are shown in Table 3. Discriminant validity was verified by selecting the two variables with the highest correlation and comparing them with the values of the AVE. The square of the correlation coefficient of “IHLC ↔ wellness,” which had the highest correlation, was 0.368, which was lower than the AVE of IHLC (0.906) and wellness (0.956), thereby securing the discriminant validity between variables.

### 2.4. Procedure and Data Analysis

This study was conducted with 844 Korean adolescents through an online questionnaire survey from January 7 to January 15, 2021. We used a questionnaire implemented in a prior study to ensure reliability and validity, and the data were analyzed using SPSS 18.0 and AMOS 18.0 software (IBM Corp., Armonk, NY, USA). The data analysis methods were as follows: (1) A frequency analysis was conducted to check the general characteristics of the subjects. (2) Cronbach’s α was used to verify the reliability of the survey tool. (3) To verify the validity of the survey tool, a CFA was conducted. (4) To verify the level of participants’ recognition of each variable, descriptive statistics were extracted. (5) Independent samples *t*-test and one-way analyses of variance (ANOVAs) were used to verify the differences between each variable according to demographic characteristics. (6) A path analysis was conducted to verify more accurately the relationship between sports participation, IHLC, and wellness.

## 3. Results

### 3.1. Descriptive Statistical Analysis

To confirm the descriptive statistics of the variables (sports participation, IHLC, and wellness) in this study, the overall and sub-dimensions were analyzed. The results of this analysis are shown in Table 4. The mean values ranged from 2.82 to 3.95, and the standard deviation values ranged from 0.537 to 1.181. Next, skewness and kurtosis were calculated. In the case of skewness, acceptable values correspond to <±3.0, and for kurtosis <±10.0, which is the basis for a univariate normality violation, so the normal distribution can generally be assessed as having met the conditions [30,31]. Through our analysis, we found that the absolute values of skewness ranged from 0.09 to 0.51 and the absolute values of kurtosis ranged from 0.15 to 2.50. These results can be evaluated as satisfying the conditions that meet the normality of the structural equation. The average age for male participants was 16.01 (±1.58 years; range 14–18 years) and 15.91 (±1.38 years; range 14–18 years) for female participants.

### 3.2. Sex Differences among Variables

An analysis of the differences in each variable by sex is shown in Table 5. Male students had higher scores in cognitive participation (M = 3.65 for male vs. M = 2.80 for female), behavioral participation (M = 3.35 for male vs. M = 2.51 for female), and affective participation (M = 3.67 for male vs. M = 3.23 for female) than female students. In the wellness section, male students had higher scores in social health and intellectual health (M = 3.35 and M = 3.78, respectively) than female students (M = 3.17 and 3.34, respectively). Similarly, male students also exhibited higher scores in emotional health (M = 3.67) than female students (M = 3.32). IHLC exhibited no sex differences.

### 3.3. Differences among Variables by School Level

An analysis of the differences in each variable by school level is shown in Table 6. Middle school students had higher cognitive participation (M = 3.29), behavioral participation (M = 3.00), affective participation (M = 3.48), intellectual health (M = 3.58), and emotional health (M = 3.54) scores than their high school counterparts (who scored M = 2.66, 2.34, 3.18, 3.31, and 3.25, respectively). IHLC exhibited no education-level differences.

### 3.4. Differences among Variables by Sports Participation Frequency

The results of analyzing the differences of each variable by frequency of sports participation per week are shown in Table 7. In the sports participation section, the cognitive participation (F = 53.222, *p* < 0.001), behavioral participation (F = 91.852, *p* < 0.001), and affective participation (F = 35.916, *p* < 0.001) variables showed statistically significant differences according to the weekly frequency of sports participation, as did IHLC (F = 16.826, *p* < 0.001). Similarly, wellness showed statistically significant differences according to the weekly frequency of sports participation in intellectual health (F = 132.108, *p* < 0.001) and emotional health (F = 105.735, *p* < 0.001) but not in social health (F = 1.851, *p* = 0.137). However, there was no difference between groups in the social health variable.

### 3.5. Differences among Variables by Sports Participation Time

Differences among variables according to the time of participation in sports are shown in Table 8. Cognitive participation (F = 78.539, *p* < 0.001), behavioral participation (F = 113.103, *p* < 0.001), and affective participation (F = 51.815, *p* < 0.001) all showed statistically significant differences according to sports participation time, as did IHLC (F = 18.247, *p* < 0.001). Additionally, social health (F = 3.580, *p* = 0.014), intellectual health (F = 115.452, *p* < 0.001), and emotional health (F = 97.557, *p* = 0.014) showed statistically significant differences according to sports participation time.

### 3.6. Differences among Variables by the Duration of Sports Participation

The differences among variables by duration of sports participation are shown in Table 9. Cognitive participation (F = 51.932, *p* < 0.001), behavioral participation (F = 85.833, *p* < 0.001), and affective participation (F = 35.833, *p* < 0.001) showed statistically significant differences according to the duration of sports participation, as did IHLC (F = 8.494, *p* < 0.001). Additionally, social health (F = 4.789, *p* = 0.003), intellectual health (F = 80.754, *p* < 0.001), and emotional health (F = 63.059, *p* = 0.014) showed statistically significant differences according to the duration of sports participation.

### 3.7. Path Analysis

In this study, three latent variables (sports participation, IHLC, and wellness) and 10 observed variables (affective participation, behavioral participation, cognitive participation, intimate behavior, emotional control, autonomous behavior, hygiene, exercise, healthy diet, and interpersonal relationships) were constructed to verify the hypothesis model. The hypothesis model’s fit was assessed to be acceptable as RMR = 0.039, NFI = 0.942, IFI = 0.951, CFI = 0.951, and RMSEA = 0.087.

The results of the verification of our research hypotheses, derived from the path analysis, are shown in Table 10. First, the path coefficient that verified the impact of sports participation on IHLC was 0.440 (*t* = 9.300), which was statistically significant. Therefore, sports participation had a positive impact on IHLC. Second, the path coefficient that verified the effect of sports participation on wellness was 0.511 (*t* = 7.311), which was statistically significant, meaning sports participation had a positive effect on wellness. Third, the path coefficient validated by IHLC on wellness was 0.539 (*t* = 7.004), which was statistically significant. Therefore, IHLC had a positive effect on wellness.

## 4. Discussion

In this section, we present a comparative analysis between the results of our study and those of previous studies.

First, examining each dependent variable in terms of the demographic variables shows partial differences. Specifically, male students had higher sports participation and wellness scores than female students (Table 7). Additionally, middle school students had higher sports participation and wellness scores than high school students (Table 8). A study by Groffik et al. [32] found that sports participation varies significantly between sex, and many prior studies have reported that health varies depending on the degree of sports participation [33]. In the aforementioned studies, sports participation inside and outside of school varied significantly, as some high school students had to concentrate on obtaining employment and passing college entrance exams [32,33]. Further, middle school students have been reported to have higher levels of sports participation than high school students. This difference in sports participation between middle and high school students has been previously reported by Shull et al. [34]. Therefore, female students and high school students should be encouraged to participate in sports to improve their wellness. However, due to social distancing, adolescents’ participation in team sports has been restricted; therefore, sports participation guidelines for maintaining minimal health in schools where sports activities are not properly conducted must be proposed and encouraged. For example, 30 min of exercise for at least five days a week, such as simple aerobic exercise, can be performed at home; alternatively, students could be encouraged to take 13,000–16,000 steps per day. These and other exercise options should be included in the guidelines to improve students’ wellness. Further, our results show that as the level of sports participation (frequency, intensity, and duration) increased, all variables, such as sports participation, IHLC, and wellness scores, increased by statistically significant amounts. This is consistent with Hui et al. [35] and Ertan and Özyol [36], who reported that knowledge of the benefits of sports participation is highly related to the level of sports participation, which can be increased through cognitive and affective participation in sports activities. In addition, Mercer et al. [37] showed that a strong IHLC was associated with high levels of sports participation. Corder et al. [38] showed that adolescents are more likely to develop obesity, cardiovascular problems, and mental problems if they lack regular and vigorous-intensity sports participation, a finding that was corroborated by Snedden et al. [39]. Therefore, in a pandemic situation where gyms, dance/fitness centers, swimming pools, and parks are being closed, it is crucial for adolescents to find PA alternatives, and habitual exercise routines should be changed to maintain PA. Especially, implementing an adapted physical training program at home during the period of the pandemic, which may well extend from weeks to months, will help decrease the negative physiological and psychological impact of sedentary behaviors [40].

Second, adolescents’ sports participation had a statistically significant positive effect on developing an IHLC. According to Marr and Wilcox’s [25] study on college students, having an IHLC positively influences sports participation by using self-efficacy and social support as mediators. Additionally, in Annesi’s study [21], participants’ IHLC was enhanced after 26 weeks of sports participation. Further, according to Towers et al. [41], developing an IHLC promotes exercise and sports participation by decreasing resistance toward exercise. Similarly, Huang et al. [42] suggested that having an IHLC promotes women’s sports participation. However, in that study, IHLC was statistically strengthened only when participants participated in sports at least four times per week, at least 60 min per session, over a period of six months or more; therefore, it can be interpreted that sporadic sports participation had a very slight or little effect on IHLC. According to the results of the present study, lack of sports participation can lead to a decrease in IHLC; for this reason, adolescents may be experiencing various forms of sports participation at home. As regular sports participation has inevitably decreased because of the pandemic [43], regular, long-term, moderate-intensity physical exercise, which can be performed in a home setting, appears to be the best option for all those affected by social distancing regulations [14].

Third, sports participation had a positive effect on adolescents’ wellness within a statistically significant range. Ohuruogu [44] investigated the main approaches to attain optimal wellness and found that sports participation and stamina can not only prevent diseases but also improve quality of life, as individuals who regularly participate in sports live longer and healthier lives. Additionally, Akbar et al. [45] found that sports participation has numerous benefits for emotional, mental, and physical health, stating that, since it is affected by family and community, maintaining healthy family relationships and systematic PA programs are important for adolescent health. In current study, it was also found that the greater the frequency, intensity, and duration of sports participation, the more the positive effects there were on all sub-dimensions of wellness.

Adolescence is an important period for the development of mental health and wellness [46,47]. However, due to the COVID-19 pandemic, contact with other people and the use of sports facilities are limited, which negatively affects adolescents’ wellness. For example, according to Qi et al.’s [48] study of 645 Chinese adults, 64.8% reported lower levels of PA and increased sedentary time during COVID-19. If this trend also applies to adolescents, it can cause indirect physical and mental problems as well as direct infection from the coronavirus [49]. Thus, it is necessary to find a way to promote sports participation because it is possible to strengthen the immune system even with moderate exercise [12].

Fourth, having an IHLC had a positive effect on adolescents’ wellness within a statistically significant range. According to a study by Xia and Ma [23], having an IHLC enhances mental health and health-related behavior by reducing the intensity of stress. Additionally, a study by Sharif et al. [22] on women with diseases suggested that to overcome diseases and actively treat them, a change from PHLC to IHLC should be made. Moreover, Steptoe and Wardle [50] found that students with a strong IHLC are more likely to exercise, eat breakfast regularly, brush their teeth daily, eat fiber, limit their salt intake, and avoid eating fatty foods. Therefore, it is believed that students with a strong IHLC are healthier because of such habits. These findings have been supported by Moshki and Ashtarian [51], who found that students with a strong IHLC are highly motivated to stay healthy.

Effective prevention of COVID-19 is highly dependent on one’s individual efforts to quarantine and socially distance. However, considering how easy it is to become exposed to the virus, individuals may develop the perception that their health is determined purely by chance, which can lead to the neglect of isolation measures. Particularly, since having an IHLC has a positive effect on mental health, strengthening it could reduce adolescent suicide rates. Therefore, it is necessary to strengthen adolescents’ IHLC and promote sports participation during social isolation situations such as those imposed by the current pandemic [52,53].

Several limitations emerged over the course of this study. First, this study examined the relationship between sports participation and wellness, using only IHLC as a variable. However, in another study, PHLC was reported to play an important role in promoting health outcomes [54]; therefore, future studies should consider other types of health loci of control, such as PHLC and CHLC, to verify the relationship between sports participation and wellness further. Second, in this study, we set sports participation, PA level, and IHLC as variables affecting the well-being of adolescents during the pandemic; however, there may be many more variables affecting wellness. Therefore, a multidimensional analysis of other variables affecting wellness should be conducted in future research. Third, this study examined the differences among variables according to the structural relationship between sports participation, IHLC, and wellness; however, there was a lack of qualitative analysis of how each variable influenced the other. Therefore, it is also necessary for future studies to investigate how and why each variable affects wellness. In particular, the mechanism by which IHLC affects sports participation and wellness should be identified. Fourth, all variables in this study were measured on a self-report scale and could have been affected by self-reporting bias. Therefore, a complementary method should be developed in future studies.

## 5. Conclusions

In juvenile educational institutions, there is a need to develop strategies to increase the wellness, sports participation, and IHLC of adolescent students, which can improve their wellness in the context of the COVID-19 pandemic.

## Figures and Tables

**Table 1:** Participants’ demographic characteristics.

Variable	Classification	Preliminary Survey		Main Survey	
		n	%	n	%
Sex	Male	106	50.0	233	36.9
	Female	106	50.0	399	63.1
Education level	Middle school	109	51.4	458	72.5
	High school	103	48.6	174	27.5
Frequency of sports participation	Never	56	26.4	189	29.9
	Once a week	81	38.2	194	30.7
	2–3 times a week	44	20.8	123	19.5
	4 times or more	31	14.6	126	19.9
Duration of individual sports sessions	None	56	26.4	179	28.3
	30 min or less	47	22.2	120	19.0
	31–60 min	55	25.9	175	27.7
	61 min or more	54	25.5	158	25.0
Duration of the current sports participation period	No participation	61	28.8	200	31.6
	3 months or less	68	32.1	171	27.1
	3–6 months	35	16.5	98	15.5
	6 months or more	48	22.6	163	25.8
Total		212	100	632	100

**Table 2:** Results of the confirmatory factor analysis.

Variables		NC	SE	Critical Ratio	*p*	Standardized Coefficient	Construct Reliability	AVE
Sports participation	Affective participation	1.000	-	-	-	0.812	0.986	0.958
	Behavioral participation	1.199	0.074	16.111	<0.001 ***	0.922		
	Cognitive participation	1.224	0.077	15.856	<0.001 ***	0.908		
Internal health locus of control	Internal #6	1.000	-	-	-	0.663	0.980	0.906
	Internal #4	0.926	0.115	8.048	<0.001 ***	0.651		
	Internal #3	1.073	0.118	9.076	<0.001 ***	0.758		
	Internal #2	1.011	0.113	8.918	<0.001 ***	0.740		
	Internal #1	0.817	0.103	7.945	<0.001 ***	0.641		
Wellness	Emotional health	1.000	-	-	-	0.867	0.984	0.956
	Intellectual health	0.870	0.075	11.548	<0.001 ***	0.733		
	Social health	0.548	0.065	8.486	<0.001 ***	0.571		

NC = non-standardized coefficient, SE = standard error, AVE = average variance extracted; *** *p* < 0.001, tested using confirmatory factor analysis.

**Table 3:** Discriminant validity verification.

Variable	Correlations between the Constructs			AVE
	Sports Participation	IHLC	Wellness	
Sports participation	1.000	-	-	0.958
Internal health locus of control (IHLC)	0.583 ***	1.000	-	0.906
Wellness	0.375 ***	0.607 ***	1.000	0.956

*** *p* < 0.001, tested using correlation analysis.

**Table 4:** Descriptive statistics (five-point Likert scale).

Variables		Mean	Standard Deviation	Skewness	Kurtosis
Sports participation	Cognitive participation	3.40	0.998	−0.13	−0.36
	Behavioral participation	2.82	1.181	0.40	−0.74
	Affective participation	3.11	1.146	0.10	−0.81
	Mean	3.11	1.042	0.29	−0.66
Internal health locus of control	Internal #1	3.98	0.793	−0.34	−0.19
	Internal #2	3.75	0.905	−0.20	−0.33
	Internal #3	3.95	0.901	−0.51	−0.15
	Internal #4	3.75	0.946	−0.39	−0.15
	Internal #6	3.68	1.021	−0.41	−0.27
	Mean	3.82	0.695	−0.05	0.19
Wellness	Social health	3.50	0.755	−0.10	−0.15
	Intellectual health	3.24	0.537	0.15	2.50
	Emotional health	3.44	0.601	−0.09	1.77
	Mean	3.41	0.488	−0.27	3.29

**Table 5:** Sex differences in all variables.

Variables	Male (*n* = 233)	Female (*n* = 399)	*t*	*p*
Cognitive participation	3.65 ± 1.07	2.80 ± 1.07	9.68	<0.001 ***
Behavioral participation	3.35 ± 1.13	2.51 ± 1.10	9.19	<0.001 ***
Affective participation	3.67 ± 0.98	3.23 ± 0.97	5.44	<0.001 ***
Internal health locus of control	3.87 ± 0.71	3.79 ± 0.68	1.341	0.180
Social health	3.35 ± 0.61	3.17 ± 0.48	3.729	<0.001 ***
Intellectual health	3.78 ± 0.76	3.34 ± 0.70	7.298	<0.001 ***
Emotional health	3.67 ± 0.79	3.32 ± 0.74	6.092	<0.001 ***

Values are mean ± standard deviation. *** *p* < 0.001, tested using an independent samples *t*-test.

**Table 6:** School-level differences in all variables.

Variables	Middle School Students (*n* = 458)	High School Students (*n* = 174)	*t*	*p*
Cognitive participation	3.29 ± 1.17	2.66 ± 0.94	7.020	<0.001 ***
Behavioral participation	3.00 ± 1.22	2.34 ± 0.91	7.406	<0.001 ***
Affective participation	3.48 ± 1.05	3.18 ± 0.82	3.759	<0.001 ***
Internal health locus of control	3.83 ± 0.72	3.80 ± 0.63	0.475	0.635
Social health	3.24 ± 0.55	3.21 ± 0.51	0.778	0.437
Intellectual health	3.58 ± 0.76	3.31 ± 0.72	4.094	<0.001 ***
Emotional health	3.54 ± 0.81	3.25 ± 0.65	4.646	<0.001 ***

Values are mean ± standard deviation. *** *p* < 0.001, tested using an independent samples *t*-test.

**Table 7:** Differences in each variable by frequency of sports participation per week.

Variable	A (*n* = 189)	B (*n* = 194)	C (*n* = 123)	D (*n* = 126)	F	*p*	Post-Hoc
Cognitive participation	2.49 ± 0.93	2.95 ± 0.87	3.60 ± 1.15	3.81 ± 1.23	53.222	<0.001 ***	A< B < C, D
Behavioral participation	2.03 ± 0.83	2.64 ± 0.81	3.40 ± 1.15	3.72 ± 1.25	91.852	<0.001 ***	A< B < C, D
Affective participation	2.91 ± 0.91	3.33 ± 0.77	3.71 ± 1.04	3.91 ± 1.03	35.916	<0.001 ***	A< B < C, D
Internal health locus of control	3.71 ± 0.73	3.73 ± 0.57	3.75 ± 0.63	4.20 ± 0.76	16.826	<0.001 ***	A, B, C < D
Social health	3.19 ± 0.61	3.21 ± 0.46	3.30 ± 0.52	3.29 ± 0.54	1.851	0.137	-
Intellectual health	2.93 ± 0.65	3.37 ± 0.50	3.94 ± 0.60	4.13 ± 0.63	132.108	<0.001 ***	A, B < C, D
Emotional health	2.97 ± 0.66	3.31 ± 0.55	3.64 ± 0.60	4.24 ± 0.75	105.735	<0.001 ***	A< B < C < D

Values are mean ± standard deviation. A: None, B: Once, C: 2–3 times, D: 4 or more *** *p* < 0.001, tested using a one-way analysis of variance.

**Table 8:** Differences among variables by sports participation time.

Variables	A (*n* = 179)	B (*n* = 120)	C (*n* = 175)	D (*n* = 158)	F	*p*	Post-Hoc
Cognitive participation	2.48 ± 0.95	2.80 ± 0.78	3.11 ± 1.09	4.06 ± 1.02	78.539	<0.001 ***	A < C < D, B < D
Behavioral participation	2.02 ± 0.83	2.52 ± 0.79	2.88 ± 1.07	3.90 ± 1.07	113.103	<0.001 ***	A < B < C < D
Affective participation	2.91 ± 0.92	3.14 ± 0.80	3.45 ± 0.90	4.08 ± 0.93	51.815	<0.001 ***	A, B < C < D
Internal health locus of control	3.72 ± 0.74	3.70 ± 0.54	3.70 ± 0.59	4.16 ± 0.75	18.247	<0.001 ***	A, B, C < D
Social health	3.18 ± 0.63	3.19 ± 0.42	3.22 ± 0.50	3.36 ± 0.53	3.580	0.014 *	A < D
Intellectual health	2.93 ± 0.67	3.31 ± 0.52	3.65 ± 0.57	4.13 ± 0.63	115.452	<0.001 ***	A< B < C < D
Emotional health	2.98 ± 0.68	3.22 ± 0.51	3.50 ± 0.59	4.14 ± 0.75	97.557	<0.001 ***	A < B < C < D

Values are mean ± standard deviation. A: None, B: Within 30 min, C: 30–60 min, D: More than 60 min. * *p* < 0.05, *** *p* < 0.001, tested using a one-way analysis of variance.

**Table 9:** Differences of each variable by the duration of sport participation.

Variables	A (*n* = 200)	B (*n* = 171)	C (*n* = 98)	D (*n* = 163)	F	*p*	Post-Hoc
Cognitive participation	2.53 ± 0.92	2.96 ± 0.91	3.34 ± 1.20	3.85 ± 1.15	51.932	<0.001 ***	A < B < C < D
Behavioral participation	2.08 ± 0.83	2.67 ± 0.91	3.11 ± 1.20	3.72 ± 1.13	85.833	<0.001 ***	A < B < C < D
Affective participation	2.94 ± 0.89	3.31 ± 0.85	3.60 ± 0.98	3.92 ± 1.01	35.833	<0.001 ***	A < B < D, A < C
Internal health locus of control	3.71 ± 0.73	3.76 ± 0.63	3.76 ± 0.52	4.05 ± 0.76	8.494	<0.001 ***	A, B, C < D
Social health	3.21 ± 0.70	3.20 ± 0.63	3.08 ± 0.44	3.33 ± 0.51	4.789	0.003 **	C < D
Intellectual health	3.00 ± 0.70	3.32 ± 0.61	3.74 ± 0.56	4.02 ± 0.65	80.754	<0.001 ***	A < B < C < D
Emotional health	3.02 ± 0.69	3.38 ± 0.66	3.56 ± 0.57	4.01 ± 0.77	63.059	<0.001 ***	A < B < C < D

Values are mean ± standard deviation. A: None, B: Within 3 months, C: 3–6 months, D: More than 6 months. ** *p* < 0.01, *** *p* < 0.001, tested using a one-way analysis of variance.

**Table 10:** Results of path analysis.

Hypothesis	Path			SC	RC	SE	CR	*p*	Verification
H1	Sports participation	→	IHLC	0.440	0.276	0.030	9.300	<0.001 ***	Accepted
H2	Sports participation	→	Wellness	0.511	0.107	0.015	7.311	<0.001 ***	Accepted
H3	Internal health locus of control (IHLC)	→	Wellness	0.539	0.179	0.026	7.004	<0.001 ***	Accepted

SC = standardized coefficient, RC = regression coefficient, SE = standard error, CR = critical ratio; *** *p* < 0.001, tested using path analysis.

## Data Availability

The data presented in this study are available on request to the authors. Some variables are restricted to preserve the anonymity of study participants.

## References

[1] Nearchou F., Flinn C., Niland R., Subramaniam S.S., Hennessy E. (2020). Exploring the Impact of COVID-19 on Mental Health Outcomes in Children and Adolescents: A Systematic Review. Int. J. Environ. Res. Public Health.

[2] O’Connor R., WetherallC K., Cleare S., McClelland H., Melson A.J., Niedzwiedz C.L., Robb K.A. (2020). Mental health and wellbeing during the COVID-19 pandemic: Longitudinal analyses of adults in the UK COVID-19 mental health & wellbeing study. Br. J. Psychiatry J. Ment. Sci..

[3] O’Connor R.C., Wetherall K., Cleare S., Eschle S., Drummond J., Ferguson E., O’Connor D.B., O’Carroll R.E. (2018). Suicide attempts and non-suicidal self-harm: National prevalence study of young adults. Br. J. Psychiatry Open.

[4] Owen N., Sparling P.B., Healy G.N., Dunstan D.W., Matthews C.E. (2010). Sedentary behavior: Emerging evidence for a new health risk. Mayo Clin. Proc..

[5] World Health Organization (2020). Coronavirus Disease (COVID-19) Advice for the Public. World Health Organization..

[6] Füzéki E., Groneberg D.A., Banzer W. (2020). Physical activity during COVID-19 induced lockdown: Recommendations. J. Occup. Med. Toxicol..

[7] Taylor S.E. (1982). Social cognition and health. Personal. Soc. Psychol. Bull..

[8] World Health Organization (1992). Basic Documents.

[9] Dunn H.L., Charles B. (1977). High-Level Wellness.

[10] Eime R.M., Young J.A., Harvey J.T., Charity M.J., Payne W.R. (2013). A systematic review of the psychological and social benefits of participation in sport for children and adolescents: Informing development of a conceptual model of health through sport. Int. J. Behav. Nutr. Phys. Act..

[11] Jansen I., LeBlanc A. (2010). Systematic review of the health benefits of physical activity and fitness in school-aged children and youth. Int. J. Behav. Nutr. Phys. Act..

[12] Füzeki E., Banzer W. (2018). Physical activity recommendations for health and beyond in currently inactive populations. Int. J. Environ. Res. Public Health.

[13] Nieman D.C., Wentz L.M. (2019). The compelling link between physical activity and the body’s defense system. J. Sport Health Sci..

[14] Onagbiye S.O., Mchiza Z.J.R., Bassett S.H., Travill A., Eijnde B.O. (2020). Novel coronavirus and regular physical activity involvement: Opinion. Afr. J. Prim. Health Care Fam. Med..

[15] Kassianos A.P., Symeou M., Ioannou M. (2016). The health locus of control concept: Factorial structure, psychometric properties and form equivalence of the multidimensional health locus of control scales. Health Psychol. Open.

[16] Bennett B.L., Goldstein C.M., Gathright E.C., Hughes J.W., Latner J.D. (2017). Internal health locus of control predicts willingness to track health behaviors online and with smartphone applications. Psychol. Health Med..

[17] Crothers L.M., Kanyongo G.Y., Kolbert J.B., Lipinski J., Kachmar S.P., Koch G.D. (2010). Job stress and locus of control in teachers: Comparisons between samples from the United States and Zimbabwe. Int. Rev. Educ..

[18] Helmer S.M., Krämer A., Mikolajczyk R.T. (2012). Health-related locus of control and health behaviour among university students in North Rhine Westphalia, Germany. BMC Res. Notes.

[19] Adame D.D., Johnson T.C., Cole S.P., Matthiasson H., Abbas M.A. (1990). Physical fitness in relation to amount of physical exercise, body image, and locus of control among college men and women. Percept. Mot. Ski..

[20] Novysedláková M., Kozáková M., Hudáková Z., Cetlová L. (2016). Promotion Health-Evaluation of the Locus of Control over Health.

[21] Annesi J.J. (2019). Effects of a group protocol on physical activity and associated changes in mood and health locus of control in adults with Parkinson disease and reduced mobility. Perm. J..

[22] Sharif S.P., Ahadzadeh A.S., Ong F.S., Naghavi N. (2020). Fear of negative appearance evaluation and attitude towards mammography: Moderating role of internal health locus of control, cancer worry and age. Health Promot. Perspect..

[23] Xia Y., Ma Z. (2020). Social integration, perceived stress, locus of control, and psychological wellbeing among Chinese emerging adult migrants: A conditional process analysis. J. Affect. Disord..

[24] Tam C.L., Bonn G., Han Y.S., Yap C.C., Wong C.P. (2016). Physical activity and its correlates among adults in malaysia: A cross-sectional descriptive study. PLoS ONE.

[25] Marr J., Wilcox S. (2015). Self-efficacy and social support mediate the relationship between internal health locus of control and health behaviors in college students. Am. J. Health Educ..

[26] Snyder E.E., Spreitzer E.E. (1983). Social Aspect of Sport.

[27] Lee S.M., Jeong H.C., So W.Y., Youn H.S. (2020). Mediating Effect of Sports Participation on the Relationship between Health Perceptions and Health Promoting Behavior in Adolescents. Int. J. Environ. Res. Public Health.

[28] Wallston K.A., Wallston B.S., Develis R. (1978). Development of the Multidimensional Health Locus of Control Scales. Health Educ. Monogr..

[29] Anspaugh D.A., Hamrick M.H., Rosato F.D. (1994). Wellness: Consepts and Appication.

[30] Kline R.B. (2011). Convergence of structural equation modeling and multilevel modeling. The SAGE Handbook of Innovation in Social Research Methods.

[31] West S.G., Finch J.F., Curran P.J. (1995). Structural Equation Models with Nonnormal Variables: Problems and Remedies.

[32] Groffik D., Fromel K., Badura P. (2020). Composition of weekly physical activity in adolescents by level of physical activity. BMC Public Health.

[33] Kudláček M., Frömel K., Jakubec L., Groffik D. (2016). Compensation for adolescents’ school mental load by physical activity on weekend days. Int. J. Environ. Res. Public Health.

[34] Shull E.R., Dowda M., Saunders R.P., McIver K., Pate R.R. (2020). Sport participation, physical activity and sedentary behavior in the transition from middle school to high school. J. Sci. Med. Sport.

[35] Hui S.S.C., Hui G.P.S., Xie Y.J. (2014). Association between Physical Activity Knowledge and Levels of Physical Activity in Chinese Adults with Type 2 Diabetes. PLoS ONE.

[36] Ertan G.A., Özyol F.C. (2020). Effects of Health-Related Knowledge and Aerobic Exercise on Lower Secondary School Students’ Obesity Awareness and Physical Activity Levels. Asian J. Educ. Train..

[37] Mercer D.A., Ditto B., Lavoie K.L., Campbell T., Arsenault A., Bacon S.L. (2018). Health Locus of Control Is Associated with Physical Activity and Other Health Behaviors in Cardiac Patients. J. Cardiopulm. Rehabil. Prev..

[38] Corder K., Sharp S.J., Atkin A.J., Andersen L.B., Cardon G., Page A., Davey R., Grøntved A., Hallal P.C., Janz K.F. (2016). Age-related patterns of vigorous-intensity physical activity in youth: The International Children’s Accelerometry Database. Prev. Med. Rep..

[39] Snedden T., Scerpella J., Kliethermes S., Norman R., Blyholder L., Sanfilippo J., McGuine T., Heiderscheit B. (2019). Sport and Physical Activity Level Impacts Health-Related Quality of Life Among Collegiate Students. Am. J. Health Promot..

[40] Hammami A., Harrabi B., Mohr M., Krustrup P. (2020). Physical activity and coronavirus disease 2019 (COVID-19): Specific recommendations for home-based physical training. Manag. Sport Leis..

[41] Towers A.J., Flett R.A., Seebeck R.F. (2005). Assessing potential barriers to exercise adoption in middle-aged men: Over-stressed, under-controlled, or just too unwell?. Int. J. Mens Health.

[42] Huang H.C., Liu L.W., Chang C.M., Hsieh H.H., Lu H.C. (2019). The effects of locus of control, agents of socialization and sport socialization situations on the sports participation of women in Taiwan. Int. J. Environ. Res. Public Health.

[43] Maugeri G., Castrogiovanni P., Battaglia G., Pippi R., D’Agata V., Palma A., Rosa M.D., Musumeci G. (2020). The impact of physical activity on psychological health during Covid-19 pandemic in Italy. Heliyon.

[44] Ohuruogu D. (2016). The contributions of physical activity and fitness to optimal health and wellness. J. Educ. Pract..

[45] Akbar L., Zuk A.M., Tsuji L.J.S. (2020). Health and wellness impacts of traditional physical activity experiences on indigenous youth: A systematic review. Int. J. Environ. Res. Public Health.

[46] Knowles G. (2017). Physical activity and mental health: Commentary on suetani et al. 2016: Common mental disorders and recent physical activity status: Findings from a national community survey. Soc. Psychiatry Psychiatr. Epidemiol..

[47] Park S.U., Ahn H., So W.Y. (2020). Developing a model of health behavior intentions and actual health behaviors of Korean male university students. J. Men’s Health.

[48] Qi M., Li P., Moyle W., Weeks B., Jones C. (2020). Physical activity, health-related quality of life, and stress among the Chinese adult population during the COVID-19 pandemic. Int. J. Environ. Res. Public Health.

[49] Brooks S.K., Webster R.K., Smith L.E., Woodland L., Wessely S., Greenberg N., Rubin G.J. (2020). The psychological impact of quarantine and how to reduce it: Rapid review of the evidence. Lancet.

[50] Steptoe A., Wardle J. (2001). Locus of control and health behaviour revisited: A multivariate analysis of young adults from 18 countries. Br. J. Psychol..

[51] Moshki M., Ashtarian H. (2010). Perceived health locus of control, self-esteem, and its relations to psychological well-being status in Iranian students. Iran. J. Public Health.

[52] Olagoke A.A., Olagoke O.O., Hughes A.M. (2021). Intention to vaccinate against the novel 2019 coronavirus disease: The role of health locus of control and religiosity. J. Relig. Health.

[53] Szlyk H.S., Berk M., Peralta A.O., Miranda R. (2020). COVID-19 takes adolescent suicide prevention to less charted territory. J. Adolesc. Health.

[54] Street R.L., Makoul G., Arora N.K., Epstein R.M. (2009). How does communication heal? Pathways linking clinician patient communication to health outcomes. Patient Educ. Couns..

